# *Hyalomma aegyptium* the dominant hard tick in tortoises *Tesdudo hermanni boettgeri* found in different regions of Albania

**DOI:** 10.1016/j.ijppaw.2022.02.002

**Published:** 2022-02-08

**Authors:** Bejo Bizhga, Bektaş Sönmez, Laurent Bardhaj, Kurtesh Sherifi, Ozan Gündemir, Sokol Duro

**Affiliations:** a Faculty of Veterinary Medicine, Agricultural University of Tirana, Tirana, 1000, Albania; b Suşehri Timur Karabal Vocational School, Sivas Cumhuriyet University, Sivas, 58600, Turkey; c Faculty of Agriculture and Veterinary, University of Prishtina “Hasan Prishtina”, Street “Bill Clinton”, Prishtine 10000, Kosovo; d Department of Anatomy, Faculty of Veterinary Medicine, Istanbul University-Cerrahpasa, Istanbul, Turkey

**Keywords:** Albania, Hermann's tortoise, *Hyalomma aegyptium*, Infestation rate, Tick prevalence

## Abstract

The purpose of this study was to estimate the level of hard tick infestation in the tortoise subspecies *Testudo hermanni boettgeri* living within the free-range hills and mountains of four regions of central and south Albania. In addition, this study showed the morphological differences of infested and non-infested tortoises in several geographic locations where tortoises are known to be infested with *Hyalomma aegyptium*, a natural carrier of different zoonotic pathogens. Thirty-six of 145 (24.8%) examined Hermann's tortoises were found to be infested with hard ticks. After the tortoises were carefully captured and controlled, a total of 67 *H. aegyptium* were collected: 47 in Berati, 11 in Ballshi and 9 in Saranda. None of the 40 tortoises in the Tirana region were found to be infested with ticks. All ticks were identified as *H. aegyptium* adults. The highest prevalence of tick infestation was in the Berati region at 49.1%, followed by the Ballshi and Saranda region by 24% and 12%, respectively. The mean infestation intensity was 1.86 *H. aegyptium* per Hermann's tortoise, and it was found that *H. aegyptium* are less common in large Hermann's tortoises. The number of *H. aegyptium* male ticks was negatively correlated with the body dimensions of Hermann's tortoises. *Hyalomma aegyptium* is the most prevalent tick in Hermann's tortoises in three regions of south Albania, and with a typical three-host life cycle in different wild and domestic animals, they may be a vector of zoonotic pathogens. Furthermore, other studies should be conducted to detect the presence of zoonotic pathogens in ticks from these regions and to estimate the risk of transmission in animals and humans.

## Highlights

• Estimation of the level of hard ticks' infestation in tortoises *Testudo hermanii boettgeri* in Albania.
• *Hyalomma aegyptium* is the only tick identified in Hermann'tortoises.
• The infestation prevalence was 24.8%.
• The mean infestation intensity was 1.86 *H. aegyptium* per Hermann's tortoise.
• *H. aegyptium* are less common in large Hermann's tortoises.

## Graphical abstract

**Figure:**
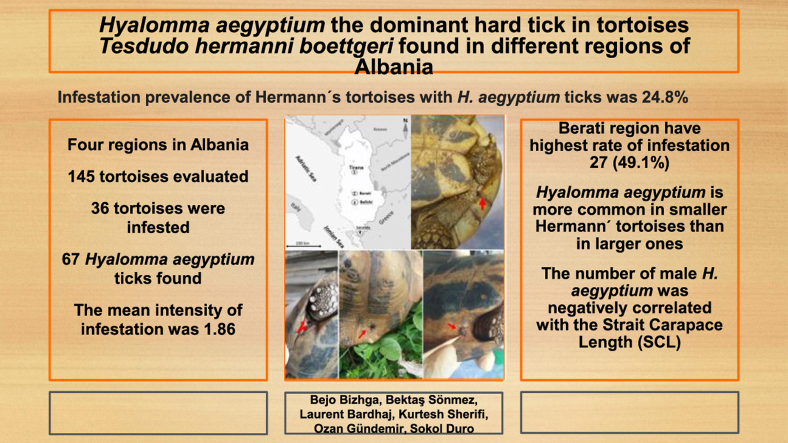

## 1 Introduction

The Hermann's tortoise (*Testudo hermanni boettgeri*), is one of the many species of Albania's fauna, which are living mostly in agricultural lands, pastures in the hills with sparse vegetation and near forest areas (Bertolero et al., 2011; Djordjević et al., 2013; Kicaj et al., 2016, Duro et al., 2021a). Albania's tortoise belongs to the subspecies Eastern Hermann's tortoise (*T. h. boettgeri*), referring to the external morphological features of the body (Fritz et al., 2006).

It is known that many tortoise species are illegally collected from nature to be kept as pets (Branch, 2008). The vast majority of these are either abandoned or confiscated by Nature Conservation units. As a result, they are usually placed under the care of veterinary clinics or zoology institutes. Their release into nature is not advised because they can transmit some diseases caused by external parasites (Cook, 2012).

Ticks are the most common external parasites in tortoises (Bertolero et al., 2011), and they differ significantly in host preference depending on the species and life stages. Ticks that do not have a chance to come into contact with a suitable host tend to feed on many types of other casual hosts, enabling the transmission and spread of various infectious diseases agents, some of which may be a zoonotic pathogen (Jongejan and Uilenberg, 2004; Kar et al., 2011). Tortoises are terrestrial reptiles that live in grassy habitats and therefore they are more likely to be infested by *Hyalomma* tick genus, which they move and actively chase for the host. Although many tick-borne pathogens in small mammals and birds have been well studied (Krawczyk et al., 2020; Roselli et al., 2020), the potential of reptiles as reservoirs of zoonotic infectious agents has often been neglected (Široký et al., 2006).

The Hermann's tortoise is listed globally as ‘near threatened’ according to the International Union for Conservation of Nature (IUCN, 2020) and as a species in need of strict protection by the Bern Convention and European Habitat Directive. Hermann's tortoises are a species present in Albania, and in certain regions they exist in considerable numbers. They often share their habitats—agricultural land, hills with rare vegetation and forest areas—with domestic and wild animals (Duro et al., 2021a). However, there is no study on their tick species prevalence in Albania, which has the implication of conferring a zoonotic pathogen carrier status on the tortoises as a wild host.

The most important ticks that are widespread in the Balkan Peninsula are the *Ixodes, Dermacentor, Haemophysalis, Rhipicephalus* and *Hyalomma* genus (Camicas et al., 1998; Estrada-Peña et al., 2004; Taylor et al., 2016). The genus *Hyalomma* is the most commonly reported tick to infest tortoises, particularly infesting the *Testudo graeca*, followed by the *Testudo horsfieldii*, while other *Testudo* species (i.e. *T. marginata)* are exceptionally mentioned (Široký et al., 2006; Uslu et al., 2019; Aouragh et al., 2020). This could be probably caused by small range of *T. marginata*, limited mostly to Greece.

*Hyalomma aegyptium* (Linnaeus, 1758) is a three-host tick and has an extremely long feeding period (Široký et al., 2011). All stages, but especially adults, are highly host-specific and feed primarily on tortoises. Occasionally *H. aegyptium* immatures are found on other animals and humans (Kar et al., 2017). *Hyalomma* is a dominant tick in tortoises from regions of the Mediterranean, Middle East, Black Sea and Central Asia. It has been confirmed that these ticks are also carriers of the Crimean-Congo haemorrhagic fever (CCHF) virus (Široký et al., 2006, 2014; Kar et al., 2020). Other zoonotic pathogens detected in *H. aegyptium* collected from tortoises in Romania were reported, including *Anaplasma phagocytophilum*, *Ehrlichia canis* and *Coxiella burnetii* (Paștiu et al., 2012).

Currently, there is no data regarding tick infestation in wild animals, specifically in tortoises from Albania. This research study enables new knowledge to be gathered on the presence of new tick species in Albanian tortoises and their potential vectorial role in the transmission of pathogens to animals and humans. The results of this study can be useful for further studies on detection of pathogens in ticks, tortoises and another domestic and wild animal.

## 2 Material and methods

### 2.1 Study area and sample collection

**Fig. 1:**
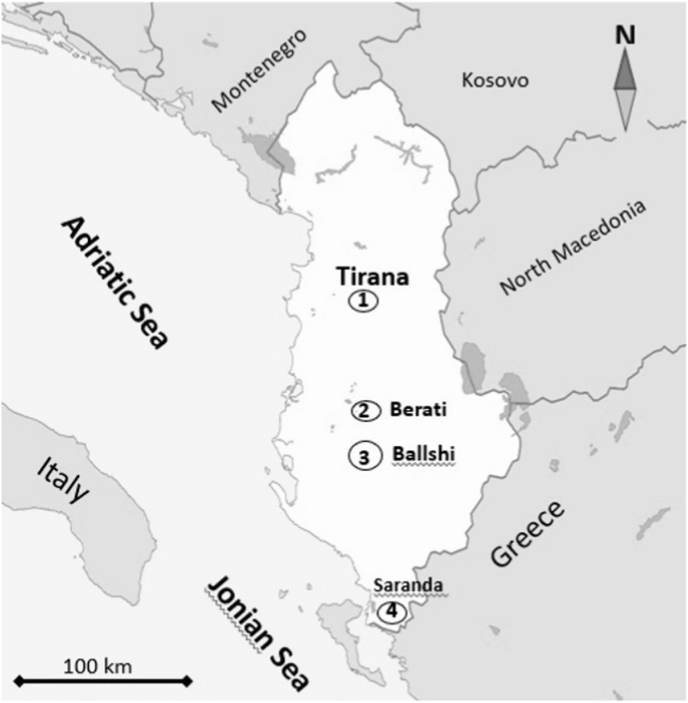
The study area from the central to south of Albania.

The study was conducted during a three-month period from April 2020 to June 2020 in four different regions of Albania, especially from central and south Albania, about 100–300 m above sea level: Tirana, Berati, Ballshi and Saranda (Fig. 1). Permission for the study was granted by the Ethical Committee of Faculty of Veterinary Medicine at the Agricultural University of Tirana (decision number:143/15.04.2020).

**Fig. 2:**
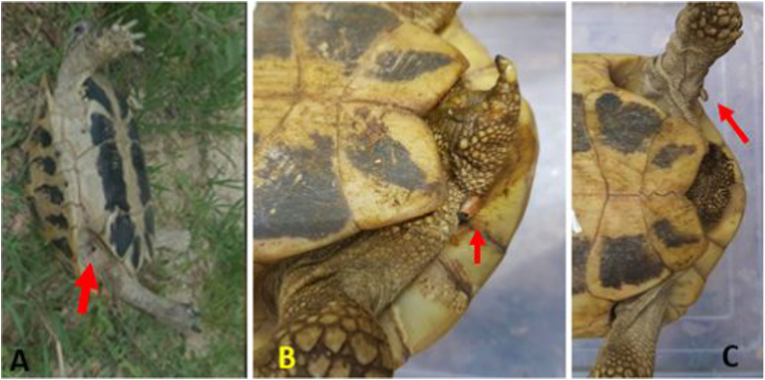
*Hyalomma* ticks in different Hermann's tortoise body part A. Tick fixed in the right inguinal region in female tortoise; B. Tick fixed in the base of the tail of female tortoise; C. Tick fixed in the left hind leg of female tortoise.

Tortoises were found randomly and captured by hand mostly in the hilly and mountainous terrain of their natural free-range habitat. They were transported in air cardboard boxes to carry out the study. Inspection of the tortoises was conducted firstly on the soft parts of the bodies starting with the neck and front legs and continuing with the region of hind legs and tail, where most of the ticks were found (Fig. 2). After careful examination of each tortoise for ticks, they were transported and sent back to the exact location from which they were taken.

### 2.2 Tick identification

After detachment of ticks from the tortoise's body with forceps, they were put in separate plastic tubes containing 70% ethanol and transported to the Laboratory of Parasitology, Veterinary Faculty of Tirana. Tick identification was performed using morphological keys according to Apanaskevich (2003) using binocular stereomicroscope (Leica S APO; 10x-80x magnification range). After species identification, the ticks were put into new plastic tubes and stored in refrigerators at temperatures of −70 °C for further studies.

### 2.3 Morphological measurements and data analysis

In order to estimate the morphological differences between infested and non-infested tortoises, we evaluated the sex and four external measurements of the Hermann's tortoise as previously reported (Duro et al., 2021a). The morphometric dimensions recorded in millimeters were: 1- Straight Carapace Length (SCL), 2- Curved Carapace Width (CCW), 3- Plastron Length (PL) and 4 -Maximal High (MH). We also recorded the tortoise weight in grams. Body condition index (CI) of the tortoise was calculated according to the standard procedures (Duro et al., 2021a).

Two parasitological indicators were calculated: infestation prevalence (%) = (number of infested tortoise × 100)/total number of examined tortoises; mean infestation intensity = number of parasites/number of infested tortoises. Prevalence (%) and mean intensity of tick infestation were counted for each region and sex separately. Tick infestation among the regions was compared with the Kruskal Wallis test. The presence of *H. aegyptium* in Hermann's tortoises related to their sex and the morphological differences between infested and non-infested tortoises were compared by Mann Whitney *U* test.

## 3 Results

In total, 145 adult Hermann's tortoises were collected from four regions of Albania and examined for the presence of tick species. Thirty-six of 145 tortoises (24.8%), 21 females (58.3%) and 15 males (41.7%), were infested with 67 *H. aegyptium* ticks. The ticks were found in different parts of the Hermann's tortoise soft body.

The number of *H. aegyptium*, the mean intensity of infestation and the prevalence by regions are given in Table 1. Hermann's tortoises in Tirana showed no tick infestation. When tortoises from the Tirana region were excluded from the dataset, the Berati region showed the highest prevalence. However, this difference is not statistically significant (p > 0.05). The mean intensity of infestation was 1.86 ± 0.99 (1–5) ticks per Hermann's tortoises, and the Berati region showed the highest rate (Table 1). Fifteen (41.6%) of the Hermann's tortoises were infested by one *H. aegyptium*, 15 (41.6%) by two, three (8.3%) by three, two (5.5%) by four and only one (2.7%) by five (Table 1).

**Table 1:** The number of infested tortoises and ticks found per tortoise in every region.

Regions	n	Infested tortoises	Prevalence (%)	Intensity of infestation	Number of ticks found per tortoise					Total ticks
					1 Tick	2 Tick	3 Tick	4 Tick	5 Tick	
Tirana	40	0	0	0	0	0	0	0	0	0
Berati	55	27	49.1	1.7	12	11	3	1	0	47
Ballshi	25	6	24	1.8	3	2	0	1	0	11
Saranda	25	3	12	3	0	2	0	0	1	9
**Total**	**145**	**36**	**24.8**	**1.86**	**15**	**15**	**3**	**2**	**1**	**67**

Sex ratio of 67 *H. aegyptium* was male biased, when 48 (71.6%) were males and 19 (28.3%) were females. The prevalence and mean intensity of *H. aegyptium* based on sex in each region are given in Table 2. It was found that the prevalence and mean intensity of infestation by *H. aegyptium* males was dominant.

**Table 2:** The prevalence and mean intensity of infestation of ticks by sex in each region.

		Male Ticks			Female Ticks		
Regions	Total Ticks	n	Prevalence (%)	Mean intensity of infestation	n	Prevalence (%)	Mean intensity of infestation
**Tirana**	0	0	0	0	0	0	0
**Berati**	47	34	72.3	1.4	13	27.6	1.1
**Ballshi**	11	8	72.7	1.3	3	27.2	1.5
**Saranda**	9	6	66.6	2	3	28.3	1
**Total**	**67**	**48**	**71.6**	**1.5**	**19**	**28.3**	**1.1**

Morphological data of infested and non-infested Hermann's tortoises are given in Table 3. The presence of *H. aegyptium* did not differ significantly between the sexes of Hermann's tortoises (p > 0.05). The carapace length, width and plastron length of infested and non-infested Hermann's tortoises differed significantly in terms of presence of *H. aegyptium* (Table 3). *Hyalomma aegyptium* are more common in smaller Hermann's tortoises than in larger ones. Any morphological sizes of the Hermann's tortoises did not correlate with the number of *H. aegyptium* (P > 0.05). However, the number of male *H. aegyptium* was negatively correlated with the SCL (n = 33, r = −0.360, p = 0.04), PL (n = 33, r = −0.402, p = 0.02) and MH (n = 33, r = −0.390, p = 0.02). However, the number of *H. aegyptium* female was not correlated with the any morphological dimension of Hermann's tortoises (p > 0.05).

**Table 3:** The descriptive statistics of infested and not infested tortoises and comparison (SCL: Straight Carapace Length, CCW: curved carapace width, PL: plastron length, MH: maximal height, CI: body condition index.

		n	Mean ± Sd	Min-Max	Z	P-level
**Weight (gr)**	Non-infested	109	1023.1 ± 358.6	242–1920	−1.898	0.058
	Infested	36	895.4 ± 379.9	160–1724		
	Total	145	989.6 ± 367.3	160–1920		
**SCL (mm)**	Non-infested	109	165.6 ± 21.9	101–216	**−2.182**	**0.029**
	Infested	36	158.9 ± 21.6	100–212		
	Total	145	163.8 ± 22.0	100–216		
**CCW (mm)**	Non-infested	109	210.8 ± 24.8	131–260	**−2.177**	**0.029**
	Infested	35	200.4 ± 27.9	135–253		
	Total	144	208.2 ± 25.9	131–260		
**PL (mm)**	Non-infested	109	146.4 ± 23.6	90–245	**−3.085**	**0.002**
	Infested	36	134.3 ± 19.5	91–185		
	Total	145	143.3 ± 23.2	90–245		
**MH (mm)**	Non-infested	109	84.2 ± 11.6	50–107	−1.090	0.276
	Infested	36	82.3 ± 11.6	40–103		
	Total	145	83.7 ± 11.6	40–107		
**CI**	Non-infested	108	0.01 ± 0.12	−0.15–0.75	−1.360	0.174
	Infested	35	−0.011 ± 0.07	−0.14–0.14		
	Total	143	0.011 ± 0.11	−0.15–0.75		

## 4 Discussion

In the present study, all ticks detected on the body of Hermann's tortoises in south Albania belong to *H. aegyptium*, with similar results found in other Mediterranean, Middle East and Black Sea countries (Široký et al., 2009, 2010; Gazyagci et al., 2010; Kalmár et al., 2015; Kheirabadi et al., 2016; Yilmaz et al., 2018; Uslu et al., 2019; Aouragh et al., 2020).

**Fig. 3:**
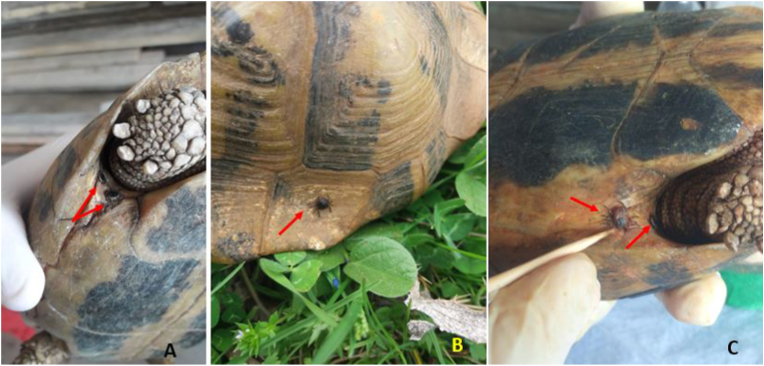
Presence of *Hyalomma* ticks on the Hermann'tortoise shell A. Two ticks fixed in the sutures of the left inguinal scute in the female tortoise; B. Tick presented in the marginal scute of the carapace on male tortoise; C. One tick fixed in the suture between plastron and inguinal scute and the second tick is found deeply in suture of right inguinal suture in female tortoise.

Interestingly, we have identified in three tortoises that the ticks were adhered to the carapace, which is not a typical location of tick infestation in tortoises (Fig. 3). Similar results related to tick infestation of adult tortoise species was reported from Italy (illegal importation of tortoises from the North Africa countries), Turkey, Malta, Algeria and Iran (Brianti et al., 2010; Kheirabadi et al., 2016; Tiar et al., 2016; Sultana Loporto et al., 2018; Kar et al., 2020).

Based on geographical locations, the results showed different prevalence and infestation rates of *H. aegyptium* in tortoises. The infestation rates vary from region to region, but they did not show a statistical differences (after Tirana samples were excluded due to a lack of ticks found in tortoises). Overall, most tortoises were infested with one to two ticks (41.7%), and in only one case, there was a tortoise infested with five ticks. Hailey et al. (1988) reported that the intensity of infestation with *H. aegyptium* in Hermann's tortoise ranged from 0.6 to 2 in Greece. Similarly, Široký et al. (2006) reported that the intensity of infestation varies between 2 and 2.3 in the Balkan countries. With an intensity of infestation of 1.86, our results are similar to those of other countries. The intensity of infestation rate in other *Testudo* species has been reported as 3.9 for *T. graeca* (Brianti et al., 2010) and 17.2 for *T. marginata* (Široký et al., 2006) in Europe. The intensity of infestation rate is lower than for other Testudo species, indicating that Hermann's tortoise is not a preferred host for the *H. aegyptium* tick.

Regarding the sex of the ticks, we found the highest percentage to be male *H. aegyptium*. This result is compatible with the previous studies (Široký et al., 2006; Aouragh et al., 2020). The higher percentage of males may be due to the fact that male ticks remain longer in tortoise hosts. Because female with an average feeding period of 25 days leave after they are fully engorged, whereas males remain on their hosts (Široký et al., 2011).

The presence of ticks did not differ between the sexes of Hermann's tortoise. The average weight of infested female and male testudines was 1054 g and 761 g, respectively. Non-infested female and male testudines weighed 1105 g and 741 g, respectively. We think that these results are related to the greater mobility found in female testudines (10 female infested Hermann's tortoises) with smaller body mass and male testudines (eight male infested Hermann's tortoises) with larger body mass. The back part of the shell in male testudines is a larger, open space compared to female testudines (Duro et al., 2021b), which is a possibility for why larger male testudines are more exposed to ticks.

In Balkan countries, no differences are found in the number of ticks between *T. marginata* sexes (Široký et al., 2006). Filippi et al. (2010) stated that the presence/absence of ticks on Hermann's tortoise did not differ between the sexes in Italy. As demonstrated in previous studies, the presence of ticks does not differ between tortoise sexes. Filippi et al. (2010) stated that the most important factor affecting the presence/absence of ticks is the thermoregulation index. Age, size, habitat and season were not deemed important factors to consider. However, it should be noted that thermoregulation may be a response to tick infestation (Filippi et al., 2010).

The results of our study, however, showed that tortoise size is one of the factors that can affect the presence of ticks. This raises the idea that Hermann's tortoises of different sizes may have different activity levels in the same habitat. It may be recommended to include factors such as age, habitat, and thermoregulation index in future studies to understand the presence/absence of ticks.

The number of ticks did not show any significant correlation with any morphological dimensions. However, the number of male ticks did show a negative correlation with body size. This is consistent with the difference between tick presence and body size. Because the presence of ticks is common in smaller tortoises, the body size of the tortoise decreases as the number of male ticks increases. This raises the idea that smaller tortoises may have higher activity levels. However, this situation was found to be different in *T. marginata*. It was reported that the body size of the *T. marginata* tortoise increased as the number of male ticks increased (Široký et al., 2006). This difference between the two species may be due to differences in size (Široký et al., 2006) and habitat preferences (Hailey et al., 1988).

In Albania, *H. aegyptium* were found to be the dominant ticks in Hermann's tortoises. However, the immature stages of *H. aegyptium* can infect humans as well, and it is known from studies in Turkey that those ticks are carriers of CCHF virus. Additional studies from Romania showed that they are vectors of *A. phagocitophylum*, *C. burnetii* and *E. canis* as well (Paștiu et al., 2012; Kar et al., 2020). This situation raises the idea that Hermann's tortoise should be considered and monitored for zoonotic diseases. Since this tortoise species shares its habitat with domestic and wild animals and includes some areas of southern Albania that are highly visited by tourists in the summer season, future studies are needed to carry out the investigations for zoonotic pathogens.

## 5 Conclusion

The study showed that only the adult *H. aegyptium* ticks were found and they are common in regions of Albania except Tirana. It is also showed that a less preferred host for the *H. aegyptium* tick of Hermann's tortoise than other *Testudo* species. *H. aegyptium* is a more common tick in Southeastern Europe (Široký et al., 2006, 2014; Kar et al., 2020), which may explain why only this tick species is found in the regions. Also its absence at northern locality, that is Tirana, could be caused by this phenomenon. Moreover, the presence of only adults may be explained by the fact that larvae and nymphs display less host-specific feeding behavior in various tetrapods (Široký et al., 2011). In addition, it has been revealed in previous studies that adult ticks prefer the *Testudo* genus tortoise as the main host (Široký et al., 2011). The results of this study, namely the presence of both the just *H. aegyptium* tick and just adults of them, were in consistent with previous studies.

This study showed that Hermann's tortoises from different regions of Albania are infested by hard ticks of *H. aegyptium* with a prevalence of 24.8% and a mean intensity of infestation of 1.86. From the 67 *H. aegyptium* ticks identified, 48 (71.6%) were males and 19 (28.3%) were females. *H. aegyptium* are more common in smaller Hermann's female tortoises and in larger male tortoises.

The fact that ticks are also present in testudines which share a common habitat with other animals and humans poses an increased risk for the transmission of various viruses, some of which may be zoonotic pathogens.

## Ethical standards

All applicable international, national, and/or institutional guidelines for the care and use of animals were followed.

## Financial Support

This research received no specific grant from any funding agency, commercial or not-for-profit sectors.

## Declaration of competing interest

The authors declare that they have no known competing financial interests or personal relationships that could have appeared to influence the work reported in this paper.
